# Fetal brain MRI atlases and datasets: A review

**DOI:** 10.1016/j.neuroimage.2024.120603

**Published:** 2024-04-06

**Authors:** Tommaso Ciceri, Luca Casartelli, Florian Montano, Stefania Conte, Letizia Squarcina, Alessandra Bertoldo, Nivedita Agarwal, Paolo Brambilla, Denis Peruzzo

**Affiliations:** a NeuroImaging Lab, Scientific Institute IRCCS Eugenio Medea, Bosisio Parini, Italy; b Department of Information Engineering, University of Padua, Padua, Italy; c Theoretical and Cognitive Neuroscience Unit, Scientific Institute IRCCS Eugenio Medea, Bosisio Parini, Italy; d Diagnostic Imaging and Neuroradiology Unit, Scientific Institute IRCCS Eugenio Medea, Bosisio Parini, Italy; e Psychology Department, State University of New York at Binghamton, New York, USA; f Department of Pathophysiology and Transplantation, University of Milan, Milan, Italy; g Padova Neuroscience Center, University of Padua, Padua, Italy; h Department of Neurosciences and Mental Health, Fondazione IRCCS Ca’ Granda Ospedale Maggiore Policlinico, Milan, Italy

**Keywords:** Fetus, Magnetic resonance imaging, Brain atlas, Brain dataset

## Abstract

Fetal brain development is a complex process involving different stages of growth and organization which are crucial for the development of brain circuits and neural connections. Fetal atlases and labeled datasets are promising tools to investigate prenatal brain development. They support the identification of atypical brain patterns, providing insights into potential early signs of clinical conditions. In a nutshell, prenatal brain imaging and post-processing via modern tools are a cutting-edge field that will significantly contribute to the advancement of our understanding of fetal development.

In this work, we first provide terminological clarification for specific terms (i.e., “brain template” and “brain atlas”), highlighting potentially misleading interpretations related to inconsistent use of terms in the literature. We discuss the major structures and neurodevelopmental milestones characterizing fetal brain ontogenesis. Our main contribution is the systematic review of 18 prenatal brain atlases and 3 datasets. We also tangentially focus on clinical, research, and ethical implications of prenatal neuroimaging.

## 1. Introduction

Quantitative analysis of brain images usually comprises some prior information in its pipeline to define a standardized coordinate system, identify specific structures, and define normative ranges. A common way to define and incorporate prior knowledge in Magnetic Resonance Imaging (MRI) studies is to define a template or an atlas. In the literature, “brain template” and “brain atlas” are often used in a partially interchangeable way, although these constructs may entail subtle but relevant differences in research and clinical practice (Dickie et al., 2017). Thus, a concise clarification of these terms is of the essence. “Brain template” (hereafter, “template”) can be used to refer to a common reference space, which is used to align and analyze images. In contrast, “brain atlas” (hereafter, “atlas”) is normally used to refer to a collection of data structured in the reference space that captures the anatomy (e.g., structural anatomy) or physiology (e.g., structural and functional connectivity) of a population. Atlases can also be defined in the temporal domain, showing how specific data change and evolve over time. This can be particularly relevant for our understanding of fetal neurodevelopment as it can provide insights into the architecture of the human brain. Therefore, in the fetal scenario, spatio-temporal atlases are the most promising tool to capture brain changes during pregnancy. To date, fetal brain atlases are defined based on the gestational age (GA) expressed in weeks.

In this work, we focused on atlases comprising structural images and some associated features or metrics (e.g., structural labels, and connectivity maps). In the literature, “atlas” is used to refer to two distinct images: the atlas reference image (i.e., MR image of the brain) and the atlas label image, which denotes anatomical structures or tissues at each brain voxel (see also Makropoulos et al., 2018). Labels allow quantitative analysis of brain volumes and shapes and may promote a better understanding of brain functioning.

Over the past decades, atlases have been the state-of-the-art approach to incorporate prior knowledge into many of the most popular segmentation algorithms (e.g., SPM-Dartel (Ashburner, 2007), FreeSurfer (Fischl et al., 2002), ANTs (Avants et al., 2011), FSL-Fast (Zhang et al., 2001)). This was and is still due to their ability to match labels from a population atlas to a new subject, thanks to a registration process that maximizes the similarity between the reference image (i.e., the atlas reference image) and the subject’s MR image. Furthermore, atlases are a prerequisite for aggregated brain development analyses, as they allow to establish correspondence between subjects. Noteworthy for our purposes, atlases continue to be a reliable tool to evaluate brain development in the prenatal period.

According to the operational definitions provided above, atlases can be classified into three main categories: “single-subject” atlases, “multi-subject” atlases, and “probabilistic” atlases. In single-subject atlases, the atlas reference image describes the anatomy of a single subject (Holmes et al., 1998), and the associated labels are usually derived from a manual process that assigns a specific label to each voxel. To overcome the limitation of relying on a single subject, it is possible to create an atlas using data from multiple subjects. Indeed, in “multi-subject” atlases, the atlas reference image is defined as the set of anatomical images from multiple subjects, while the atlas label image refers to the collection of computed labels for each subject. Hereafter, we refer to such “multi--subject” atlases also as “datasets”. In contrast, probabilistic atlases derive their atlas reference image by combining images from multiple subjects, thus reflecting the anatomical variability in the population anatomy (Mazziotta et al., 2001; Irfanoglu et al., 2022). In this scenario, automatic segmentation methods are typically used to assign each voxel a probability for a given label.

Construction of fetal MRI brain atlases versus postnatal atlases faces two main challenges. First, fetal MRI is itself a challenging task due to the size of the brain, the mother’s body in the scanner (sometimes also siblings), and movements of the fetus (Uus et al., 2023b). The most common acquisition protocol for fetal MRI involves acquisition of fast 2D sequences, including a few slices with a high planar resolution but also large thickness (Ciceri et al., 2023). Acquired images can have motion-induced artifacts (e.g., inconsistencies between consecutive slices) and an acquisition plane different from the standard ones (i.e., axial, coronal, sagittal). Second, during the prenatal period, the brain is extremely plastic and undergoes impressive growth (Garcia et al., 2018a). Thus, it is necessary to employ specific brain atlases for very narrow temporal windows (i.e., in the range of weeks) (Evans et al. 2012).

Focusing on the prenatal scenario, to the best of our knowledge only two works provided rigorous reviews of brain atlases (Makropoulos et al., 2018; Oishi et al., 2019). Although Makropoulos and colleagues primarily aimed to provide a comprehensive review of automatic brain segmentation techniques in the prenatal and neonatal periods, they also screened available atlases and found two probabilistic atlases for the fetal domain. The following year, Oishi and colleagues carried out similar study also postnatally, until 72 months. Three probabilistic atlases were thus reported for the prenatal period. Since then, several published studies have addressed the problem of defining templates and atlases for fetal MRI.

In the current work, we aim to provide an up-to-date comprehensive review of recent findings in this challenging field of research. We specifically focused on the prenatal period and screened the recently introduced MRI atlases as scientific and clinical interest is increasing and neuroimaging techniques (e.g., diffusion MRI, Karimi et al., 2021) made advances in exploring fetal development. We also report publicly available links as data availability is crucial for the development of artificial intelligence (AI)-based algorithms capable to address the broad spectrum of fetal brain changes and disease-related anatomical alterations. AI tools for fetal neuroimaging are probably the most vibrant and promising challenge in the field. These and other advances in fetal imaging can be a potential game changer for future research and clinical applications, but such an approach may pose significant ethical concerns.

### 1.1. Prenatal brain development framework

**Fig. 1.**
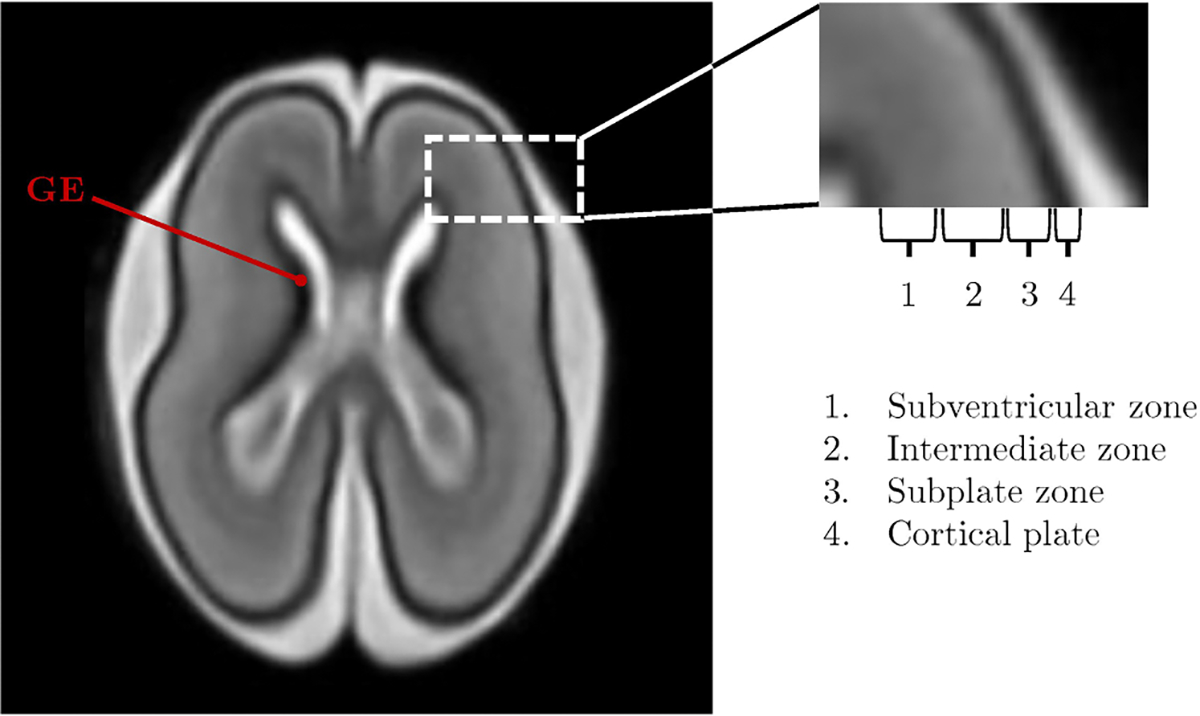
T2w image showing the brain structure of a 21-week fetus. The image zoom depicts the fetal telencephalic wall and its sub-structures: subventricular zone, intermediate zone, subplate zone, and cortical plate. The fetal brain depicted here is adapted from the publicly available atlas by Uus et al. (2023). GE: ganglionic eminence.

During pregnancy, fetal brain undergoes significant development of its multiple brain structures, characterized by cellular proliferation, migration, and network formation (Serati et al., 2019). Fetal brain MRI has the potential to monitor changes in size and morphology of different brain regions. Some regions may/should also disappear over time. For example, an interesting transitory structure is the ganglionic eminence (GE) (Fig. 1). It is a proliferative structure of the ventral telencephalon of the fetal brain that appears during the 5th week post-conception and disappears within the 35th week of gestation (Boitor-Borza et al., 2021), and it contains precursor neurons of basal ganglia, thalami, amygdala and cerebral cortex interneurons (Righini et al., 2013).

Particularly noteworthy is necessary for the fetal telencephalic wall with its sub-structures. It has a stratified structure characterized -from the inside out-by a ventricular and subventricular zone, intermediate zone, subplate, and cortical plate (CP, Huang et al., 2010) (Fig. 1). From the ventricular and subventricular zone, neurons proliferate and migrate radially towards the brain surface, forming several brain layers such as the CP and subplate, which will eventually mature into neocortex (Barkovich et al., 2012). The subplate is a transient cerebral wall compartment, in which progenitor cells and afferents of the thalamus and cerebral areas segregate and grow. It is one of the first areas to develop in the human cerebral cortex. The subplate has been described as a cortical amplifier that coordinates cortical activity. Sensitive growth and migration windows have crucial consequences for cognitive functioning (Serati et al., 2019). It has been hypothesized that alterations in the early subplate synaptic circuitry due to genetic load may be involved in the pathophysiology of neuropsychiatric and neurodevelopmental conditions such as schizophrenia and autism (Harkin et al., 2017). The CP undergoes a process of progressive folding of its surface, with multiple sulci and gyri forming during fetal life, in particular during the second and third trimesters, with a linear increase as GA increases (Garcia et al., 2018; Xu et al., 2022). As gyrification progresses, there is a gradual expansion of the surface area, with exponential growth with as GA increases. Conversely, cortical thickness follows a biphasic progression, which first increases and then decreases with GA (Xu et al., 2022).

Several insults occurring during pregnancy can alter this complex mechanism of brain cellular organization, resulting in brain damage. Therefore, quantitative characterization of the aforementioned structures is an interesting proxy to evaluate the growth of the fetal brain and, hopefully, promptly detect early deviations from the normal developmental path. In this scenario, spatio-temporal atlases of the fetal brain are an useful tool for the assessment of potential derailments or anomalies in the prenatal neurodevelopment trajectories, and provide a needful reference space for population-based analyses.

## 2. Methods

A literature search was performed in September 2023 and then repeated in January 2024 by the first author (TC) using appropriate search terms related to *“fetus”*, “*brain*”, *“MRI”,* and *“atlas”* or “*template*” or “*dataset*” (see Supplementary Material 1) in the *PubMed* bibliographic database. Furthermore, a manual search was performed by using the Google search engine to identify all evidence relevant to our research questions.

All included articles were peer-reviewed in English, without any predefined date limits or sample size restrictions.

The exclusion criteria for the current review applied to retrieved articles were:

- studies not including in-vivo fetal participants;
- sample data which was not MRI-related (i.e., ultrasound imaging);
- sample data which was not brain-related;
- articles not including what we refer to atlas reference images or atlas label images.

Case reports, review articles, and opinion papers were excluded from the results, although their reference lists were manually assessed for important additional references.

In the following section, we present our review findings according to this specific dichotomy: fetal brain MRI probabilistic atlases, and fetal brain MRI datasets.

## 3. Results

**Fig. 2.**
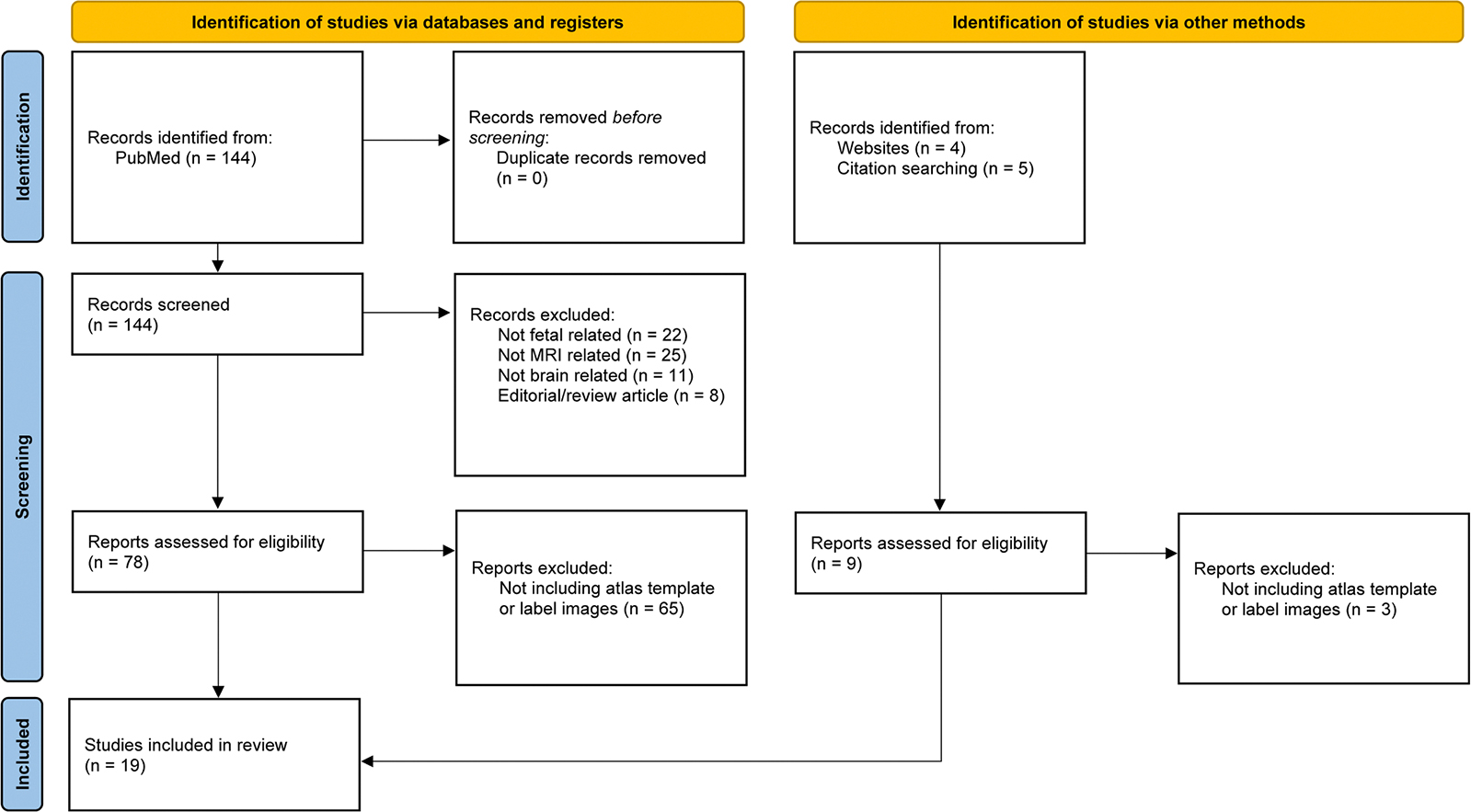
PRISMA flow diagram. It reports the study selection process used in this review.

Our databases search strategy yielded 144 studies. After checking for duplicates and screening titles and abstracts, we identified 87 potential articles, 9 of which resulted from a more extensive manual search using the Google search engine. Nineteen studies were included in this review based on inclusion and exclusion criteria. The study selection process is illustrated in Fig. 2.

**Fig. 3.**
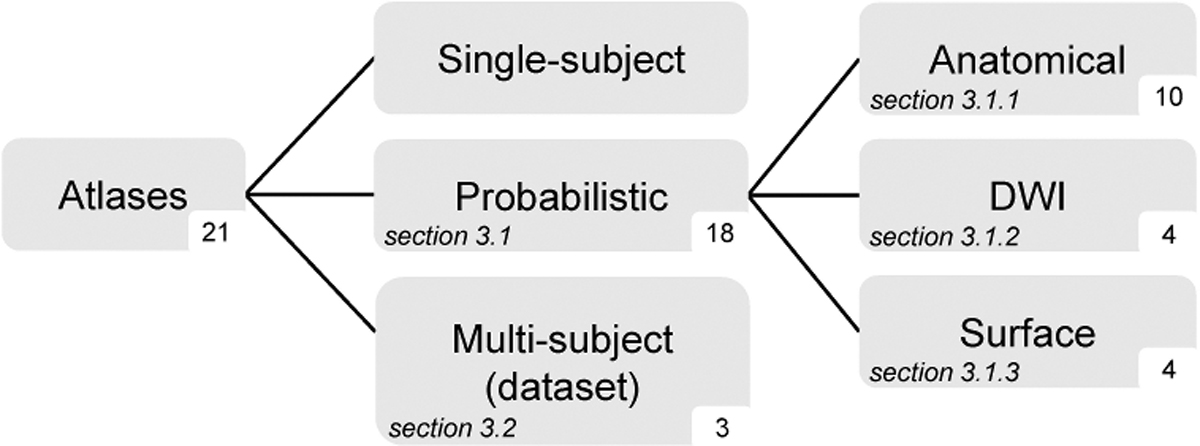
Categorization of atlases. The number of atlases per category is reported at the bottom right of each box. No single-subject atlas has been identified. Probabilistic atlases are categorized on the basis of the reference image used to characterize the brain anatomy (i.e., anatomical, diffusion, and surface images). DWI: diffusion weighted imaging.

We identified 21 atlases (Fig. 3), 15 of which are accessible online. Notably, 18 brain atlases are categorized as probabilistic atlases, whereas the remaining 3 atlases are classified as datasets (i.e., multi-subject atlases). Furthermore, one study provided both a probabilistic atlas and a dataset (Urru et al., 2023).

**Fig. 4.**
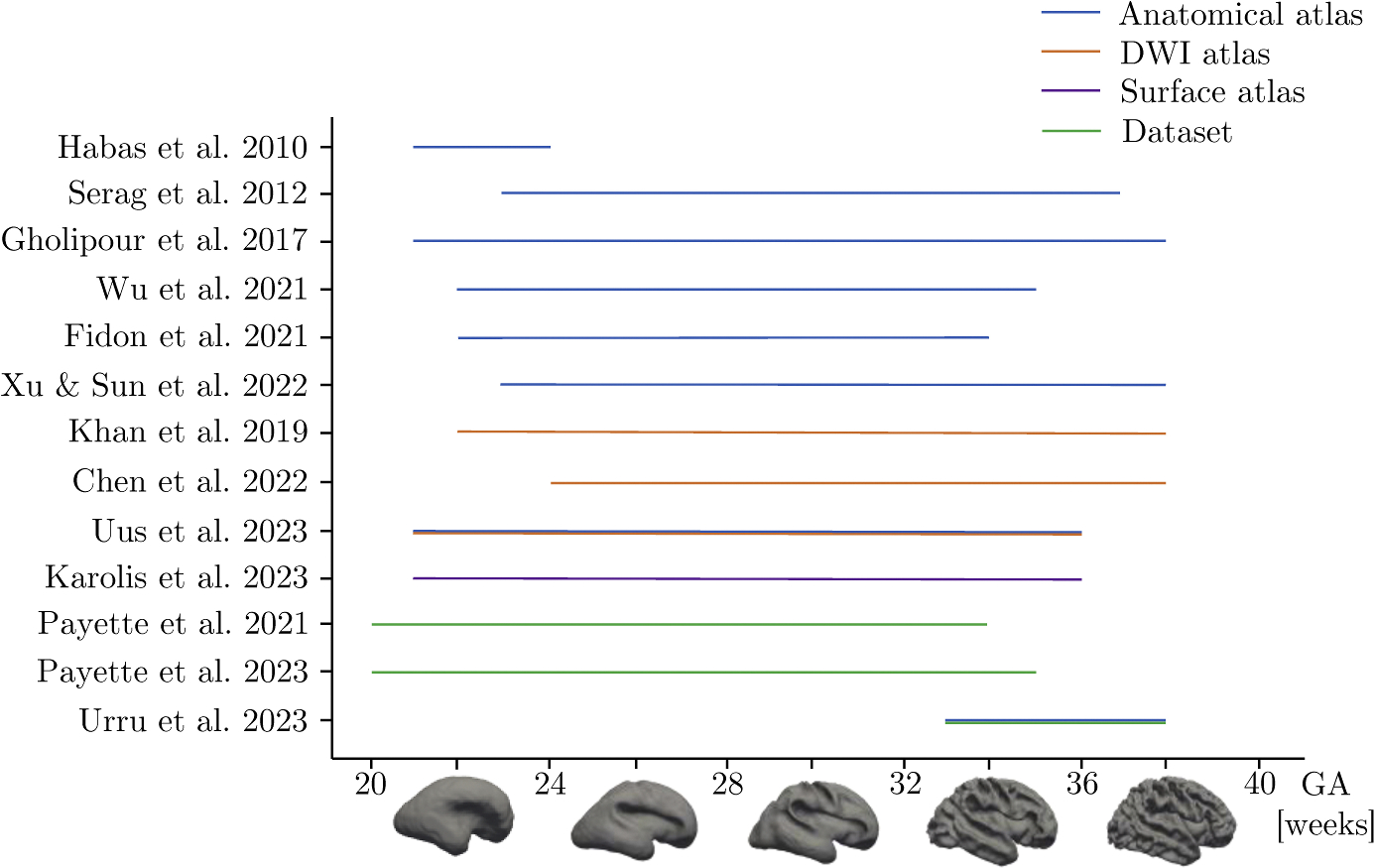
Gestational age domain of publicly available fetal MRI atlases or datasets. The graph describes gestational age, in terms of weeks, covered by each fetal MRI atlas or datasets included in this review. Examples of normal appearing fetal cortical surfaces at different GAs are reported along the x-axis. DWI: diffusion weighted imaging.

A crucial aspect to consider when dealing with a fetal atlas is its gestational domain, i.e., the range of GAs that are covered by the atlas. Fetal atlases with wide gestational domains usually contain various sub-atlases that cover small portions of the overall domain. In Fig. 4, we reported GA, in terms of weeks, covered by the publicly available fetal MRI atlases and datasets.

**Fig. 5.**
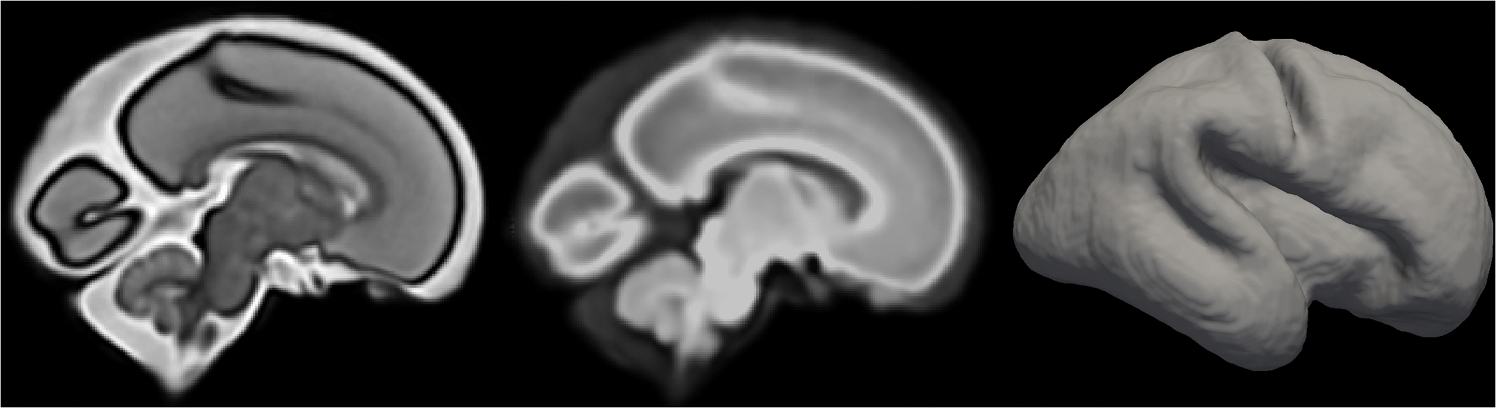
Example of different atlas reference images in a fetal brain atlas. From left to right: structural anatomical, structural diffusion, and surface images of a 28-week fetus. The structural anatomical and structural diffusion atlas reference images are generated from the publicly available atlas of Uus et al. (2023). The atlas reference image of the surface atlas depicted shows the cortical plate, and is generated via MATLAB from the previously quoted Uus et al. (2023) publicly available atlas.

The MRI template images included in atlases and datasets are 3D high-resolution reconstructions of the fetal brain. As previously described, the standard fetal brain acquisition protocol includes ultrafast 2D sequences given their lower susceptibility to fetal movement and higher SNR (Glenn, 2010; Gholipour et al., 2014), resulting in voxels with an anisotropic size ratio. However, thanks to advanced super-resolution reconstruction algorithms such as NiftyMIC^1^ (Ebner et al., 2020), MIALSRTK^2^ (Tourbier et al., 2015), SVRTK^3^ (Kuklisova et al., 2012), and NeSVoR^4^ (Xu et al., 2023) these multiple 2D fetal scans can be combined to generate high-resolution fetal brain reconstructions with isotropic voxel size. Notably, datasets typically provide as atlas reference images only the 3D high-resolution reconstructed images for each subject. By contrast, probabilistic atlases include different types of high-resolution 3D reconstructions as atlas reference images, according to their intended purpose and modality: anatomical images, diffusion images, and surfaces (Fig. 5).

### 3.1. Probabilistic MRI atlases of the fetal brain

Among the different fetal brain MRI probabilistic atlases, we can identify three distinct atlases based on the structure of the atlas reference image or acquisition modality: anatomical atlases, Diffusion Weighted Imaging (DWI) atlases, and surface atlases.

**Fig. 6.**
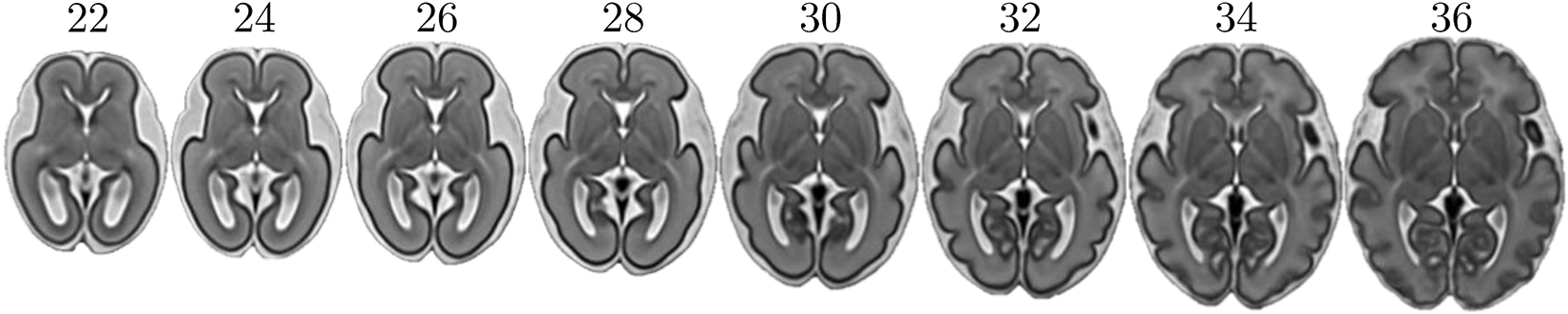
T2-weighted images of the fetal brain covering weeks 22 to 36. The maps are generated from the Uus et al. (2023) publicly available atlas.

**Fig. 7.**
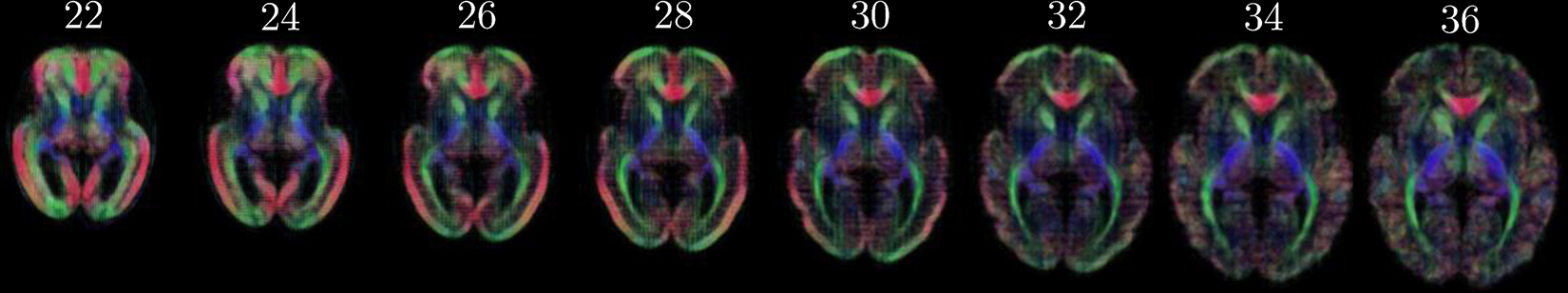
Fiber Orientation Distribution (FOD) maps of the fetal brain covering 22 to 36 weeks. Maps are generated from the Uus et al. (2023) publicly available atlas.

**Fig. 8.**
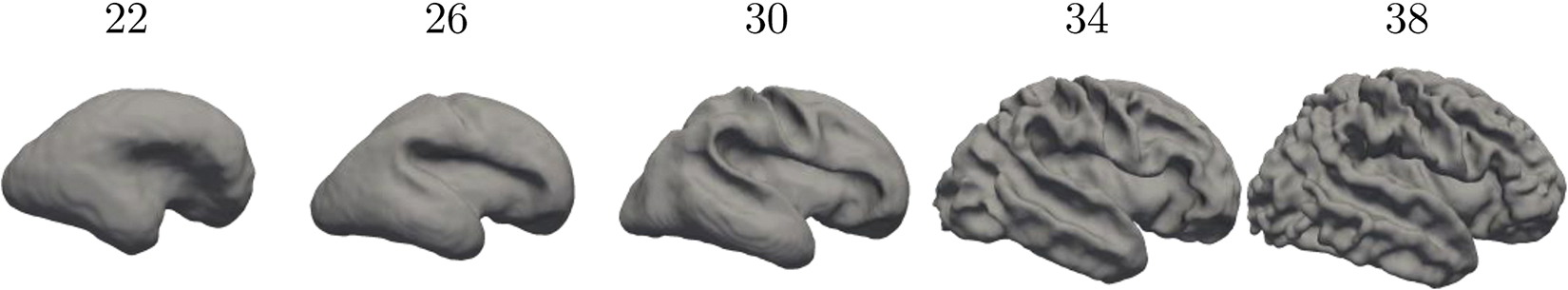
Cortical surface of the fetal brain covering 22 to 38 weeks. The surfaces depicted in the figure are generated from Gholipour et al. (2017) publicly available atlas.

We identified 18 probabilistic atlases of the fetal brain, 12 which are publicly available. The majority of the publicly available atlases (*N* = 8) provided, as atlas reference image, a T2-weighted (T2w) volumetric high-resolution image (Fig. 6), which is associated with an atlas label image defining some structures of interest. They are usually employed to identify structures on new subjects. Other publicly available atlases (*N* = 3) are based on DWI images and provide, as atlas reference image, several reconstructed parametric maps derived from widely used Diffusion Tensor Imaging (DTI) (Basser et al., 1994) model, such as Fractional Anisotropy (FA), color-coded FA (cFA), Mean Diffusivity (MD), Radial Diffusivity (RD), Axial Diffusivity (AD) and Fiber Orientation Distribution (FOD) (Fig. 7). These parametric maps use the microscopic movement of water molecules to investigate the arrangement of the developing complex fiber structure of the brain (De Luca et al., 2021), thus they can be directly compared to a new subject. One of them also provides an atlas label image. The third type of atlases (i.e., surface atlases) provided, as atlas reference image, meshes representing the surfaces of fetal brain structures (Fig. 8). These are usually derived from the high-resolution T2w volumetric image and are typically developed to better study the age-related variability of the highly convoluted cerebral cortex. Several biometric measurements (e.g., surface area, cortical thickness, and cortical folding/gyrification) can be derived from the cerebral cortical surface to comprehensively provide various detailed aspects of the cerebral cortex characterization across the GA (Li et al., 2015). In addition, surface atlases could be used to align or map surfaces of complex or irregular anatomical structures obtained from different MRI scans, providing a more accurate match (Cachia et al., 2003).

Table 1 shows an overview of publicly available fetal brain MRI atlases grouped by their intended purpose and modality.

#### 3.1.1. Anatomical atlases

Habas et al. (2010) constructed the first fetal brain spatio-temporal atlas from 20 reconstructed (voxel size of 0.5 mm isotropic) normal-appearing brains. The fetal atlas is defined for each week in the range of 21–24 GA and consists of age-specific T2w atlas reference images and tissue probability label images of the developing cortical gray matter (cGM), the developing white matter (WM), the germinal matrix, and the lateral ventricles (LV). To create the atlas, they performed a groupwise registration of tissue label maps extracted from fixed manual segmentation between subjects and then modeled the changes in MR intensity, tissue probability, and shape of fetal brains with a quadratic polynomial. This atlas is a first attempt to provide a standardized framework for comparing and analyzing fetal brain MR images. It is not directly accessible, but it can be shared by the authors upon request and by a data-sharing agreement.

Serag et al. (2012) derived a spatio-temporal brain atlas from 80 reconstructed (voxel size of 1.18 mm isotropic) fetuses with normal-appearing brains. The fetal atlas is defined for each week in the range of 23–37 GAs and consists of age-specific T2w atlas reference images and tissue probability label images of the cortex, the hemispheres, the cerebrospinal fluid (CSF), and the LV. The tissue probability images are generated by performing manual segmentation for each subject. The method used for the spatio-temporal atlas construction relies on a non-rigid registration approach to eliminate the bias between all pairs of images and then on an adaptive kernel regression algorithm (Nadaraya et al., 1964) to produce anatomical template images for each week. Notably, use of a time-varying kernel width allows to overcome variations in subject distribution across ages. Such an approach is the main specificity of this atlas, considering that it retains a consistent level of detail at every time point.

Dittrich et al. (2014) introduced a spatio-temporal latent atlas of the fetal brain from 32 reconstructed (voxel size between 0.78 mm isotropic and 0.9 mm isotropic) fetuses in healthy pregnancies. The fetal atlas is defined for each week in the range of 20–30 GAs and consists of age-specific T2w atlas reference images equipped with the label map of the LV structure. However, the main novelty of this study rests in the semi-supervised methods allowing to segment the brain from partially annotated subjects, thus reducing the time required for manual annotations. As the core of the manuscript was the atlas construction method, the atlas itself is not publicly available.

Gholipour et al. (2017) derived a spatio-temporal atlas of the fetal brain from 81 reconstructed (voxel resolution of 1 mm isotropic) fetuses in healthy pregnancies. The updated version of the fetal atlas, released in February 2018, is defined for each week in the range of 21–38 GAs and consists of age-specific T2w template and label images of 124 brain structures (manually refined from ALBERTS atlases (Gousias et al., 2012 and 2013) and propagated from the higher GAs to the lower GAs), including cGM, WM, deep GM (dGM), CSF, LV, brainstem (BS), cerebellum (CB), and etc. In addition, the atlas, which covers 21–30 GAs, includes labels for the developing WM layers such as the subplate, the intermediate zone, and the ventricular zone. Differently from previous works (Habas et al., 2010; Serag et al., 2012; Dittrich et al., 2014), in which atlas construction relied upon manual segmentations of original data, Gholipour and colleagues focused on the construction of a sharp deformable spatio-temporal atlas to facilitate use of a probabilistic label fusion approach for atlas labeling and segmentation. They integrated a kernel regression accounting for GA with a symmetric diffeomorphic deformable spatial registration (Avants et al., 2008) to build a detailed atlas that is an unbiased average representative of the anatomy of all key GAs. The atlas marks a crucial advancement in brain studies and clinical assessments (Mufti et al., 2023; Stuempflen et al., 2023; Machado-Rivas et al., 2023) as it is included in several super-resolution reconstruction algorithms such as NiftyMIC (Ebner et al., 2020), MIALSRTK (Tourbier et al., 2015), and NeSVoR (Xu et al., 2023). It is used to define a standard radiological anatomical space for the reconstruction of high-resolution isotropic images of new subjects.

Li et al. (2021) introduced a spatio-temporal atlas of the fetal brain from 35 reconstructed (voxel resolution of 0.8 mm isotropic) fetuses in healthy pregnancies recruited from the Chinese population. The fetal atlas is defined at 2-week interval in the range of 23–36 GAs and consists of age-specific T2w templates, with a brain tissue mask (i.e., without CSF) defined on the basis of the segmentation tool proposed in Makropoulos et al. (2014) followed by a manual correction. The atlas reference images were generated using an iterative deformable registration approach. The algorithm selects five fetal brains in a temporal interval of 2 weeks centered on the desired target GA and performs a multi-stage registration (affine, affine + non-linear) to compute a single template that is associated with the target GA. Differently from other templates, the authors did not define any ROIs but performed a voxel-based analysis. This atlas is a first attempt to focus on a specific population, i.e. the Chinese one. However, the atlas is not directly accessible, even though it can be shared by the authors upon request and by a data-sharing agreement.

Wu et al. (2021) derived a spatio-temporal atlas of the fetal brain from 89 reconstructed (voxel resolution of 0.8 mm isotropic) fetuses in healthy pregnancies recruited from the Chinese population. The atlas is defined for each week in the range of 21–35 GAs and consists of age-specific T2w atlas reference images and label images of 124 brain structures as introduced by Gholipour et al. (2017). These age-specific volumetric fetal brain templates were generated using the ANTs unbiased group-wise registration algorithm (Avants et al., 2008), and their labels were derived by a propagation approach from the Gholipour et al. (2017) atlas, followed by a manual refinement. Differently from the original manuscript, the available templates range from 22 to 35 GAs. This atlas is the first publicly available attempt to characterize prenatal brain development in the Chinese population.

Fidon et al. (2022) derived the first spatio-temporal atlas of the fetal brain from 37 reconstructed (voxel resolution of 0.8 mm isotropic) Spina Bifida Aperta (SBA) fetuses. The fetal atlas is defined for each week in the range of 21–34 GAs and consists of:

- a reconstructed T2w template image and its brain mask label image;
- a tissue label image for WM, ventricular system, CB, extra-axial CSF, cGM, dGM, BS, and corpus callosum (CC);
- an annotation of seven anatomical landmarks, including the anterior horns of the LV, the posterior tectum plate, the junctions between the CB and the BS, and the dGM borders at the foramen of Monro.

The pipeline followed for atlas construction relies on an initialization step based on the weighted generalized Procrustes (Gower et al., 1975) method that uses the anatomical landmark annotations only to linearly align volumes, followed by a time-weighted volume average; a refinement step to improve the image sharpness of the intermediate atlas by non-linearly registering (Modat et al., 2014) all the fetal brain reconstructions to the intermediate volumes and computing new weighted volume average; and finally a postprocessing step to manually correct the obtained segmentations of the atlas. The proposed atlas is an example of a clinical population atlas (i.e., SBA fetuses) developed to support clinical practice (i.e., the peri-surgical SBA phases). Furthermore, it provides tailored priors for the development of ad-hoc DL-based segmentation methods.

Xu & Sun et al. (2022) derived a spatio-temporal atlas of the fetal brain from 90 reconstructed (voxel resolution of 0.8 mm isotropic) fetuses in healthy pregnancies recruited from the Chinese population. The atlas is defined for each week in the range of 23–38 GAs and consists of age-specific T2w template images and label images of 85 brain structures, including cortical and dGM, WM, CSF, LV, BS, CB, hippocampus (Hp), and amygdala (defined using the Draw-EM algorithm of Makropoulos et al. (2014)). The atlas was generated using adaptive kernel regression (Nadaraya et al., 1964) to regroup subjects according to their GA, followed by a group-wise registration (Avants et al., 2008) pipeline with pairwise initialization to generate age-specific atlas. Contrary to previously introduced atlases focused on the Chinese population (Li et al., 2021; Wu et al., 2021), this study introduces several novelties to enhance comparability with other existing atlases. These include the use of a 3T scanner rather than 1.5T, higher resolution images (i.e., slice thickness < 2 mm), and a wider GA range.

In Urru et al. (2023) and colleagues exploited the data available from Gholipour et al. (2017) to build a new version of this atlas. This new atlas was generated by registering both the age-specific T2w template images and their label images to the corresponding neonatal temporal images of Serag et al. (2012). After performing registration for every time point, label correspondence was performed by assigning the most frequent or probable class. Therefore, the 124 structure labels, defined by Gholipour et al. (2017), were standardized across the whole GA range (21–38 GAs) in 9 constant tissues labels, which also include labels defining the different WM layers. They included this new version of the atlas in a unified framework performing brain segmentation both from fetal and neonatal brain images. This approach addresses the lack of a single tool for perinatal brain development analysis. In addition, they also introduced a publicly available dataset specifically designed to aid in the segmentation process (see Section 3.2 for a more detailed description).

#### 3.1.2. DWI atlases

Khan et al. (2019) derived the first atlas of the fetal brain based on the diffusion tensor images (DTI) of 67 fetuses in healthy pregnancies. Following the same approach of the T2w images, the DWI data were reconstructed at a higher resolution, with a final voxel size of 0.75 mm isotropic. The fetal atlas is defined for each week in the range of 22–38 GAs, and it is an extension of the Gholipour et al. (2017) atlas. It comes with age-specific DTI template images (estimated from a maximum of 2 vol with *b* = 0 s/mm^2^ and 12 vol with *b* = 500 s/mm^2^) and their estimated label images of FA, cFA, and MD. As in Gholipour et al. (2017), the spatio-temporal dMRI atlas was developed based on a kernel-regression strategy using tensor-based registration for accurate alignment of WM structures to produce an unbiased age-regressed template at any given age point. This DTI atlas enables researchers to explore new frontiers in fetal brain imaging analysis, detecting major neuronal fiber bundle pathways, and characterizing fetal brain reorganization that occurs in utero. In 2022, Calixto et al. expanded this atlas manually, labeling 14 regions of interest (CP, subplate, intermediate zone, GE, anterior and posterior limbs of the internal capsule, genu, body, and splenium of the CC, Hp, lentiform nucleus (Ln), thalamus (Th), BS, and CB) defined for each week in the range of 23–30 GAs. This new set of labels was used to characterize the different structures in the DWI images but is not publicly available.

Chen et al. (2022) derived a spatio-temporal DTI atlas of the fetal brain from 89 reconstructed (voxel resolution of 1.2 mm isotropic) fetuses in healthy pregnancies. The fetal atlas is defined for each week in the range of 24–38 GAs and comes with the label images of FA, cFA, MD, RD, and AD estimated from the age-specific DTI template images (obtained from 30 vol with *b* = 600 s/mm^2^). Furthermore, label images include FOD maps within a voxel for each week in the range of 24–26 GAs. Differently from Khan et al. (2019), the spatio-temporal DTI atlas was developed by a kernel-regression strategy that uses FOD-based registration versus tensor-based ones for a more accurate alignment of structures to produce an unbiased age-regressed template at any given age point. Instead of taking a direct averaging after rigidly aligning subject images, the authors used pair-wise registrations with shape updates in the initialization of atlas generation to avoid bias from individual brains. This atlas presents reconstructed images at higher resolution (1.2 mm isotropic) compared to Khan et al. (2019) reconstructions (0.75 mm isotropic), and it is defined at a narrow GA range (24–38 weeks).

Uus et al. (2023) introduced a multi-channel spatio-temporal MRI atlas of normal fetal brain development as a part of the developing Human Connectome Project (dHCP project). The cohort selected for atlas construction includes 187 fetuses without reported anomalies and with good MR image quality. In detail, fetal brains were reconstructed with a voxel resolution of 0.5 mm isotropic. The atlas is defined for each week in the range of 21–36 GAs and consists of 4 channels: structural (T2w, T1w) and diffusion (FA, MD, RD, average DWI *b* = 1000s/mm^2^, FOD) MRI channels, tissue segmentation and label description channels. The spatio-temporal atlas was constructed using the MIRTK toolbox, similar to the dHCP neonatal atlas based on (Schuh et al., 2018) with multi-channel (T2w+cortex) guided registration. The multi-label brain tissue segmentation consists of 19 ROIs with separate R/L structures based on the fetal brain histology atlases (Bayer and Altman, 2003 & 2005); the segmentation map was created using a combination of semi-manual refinement of the optimized dHCP Draw-EM neonatal pipeline segmentations (Makropoulos et al., 2018b) and registration between the atlas timepoints. It is a particularly comprehensive mapping of fetal brain development considering that it is based on the highest number of fetuses (187) in a multi-channel-based approach. Moreover, reconstructed images show a high level of resolution (0.5 mm isotropic). However, it is defined at a narrower GA range compared to other atlases.

#### 3.1.3. Surface atlases

Clouchoux et al. (2012) constructed a spatio-temporal surface atlas of the cortical plate from 12 reconstructed (isotropic voxel resolution of 1 mm isotropic) fetuses in healthy pregnancies. The atlas is defined on four GA ranges: 25–28, 28–30, 30–32, and 32–35 and includes cortical plate surfaces equipped with the probability template images of sulci location (insula, central sulcus, inferior frontal sulcus, superior temporal sulcus, postcentral sulcus, superior frontal sulcus, intra-parietal fissure, precentral sulcus, and inferior temporal sulcus) for each week range. Its construction relied on an iterative registration of the cortical plate surfaces, as described in Lyttelton et al. (2007), followed by an averaged surface computation. The resulting atlas was used to describe the fetal folding pattern and the gyrification process during pregnancy. It is not made publicly available.

Wright et al. (2015) constructed a spatio-temporal surface atlas of the developing cerebral cortex from 80 reconstructed (voxel resolution of 1 mm isotropic) fetuses in healthy pregnancies. The spatio-temporal surface atlas is defined for each week in the range of 23–37 GAs and includes the cortical GM/WM surface template image and its 19 segmentation images that were propagated from a neonatal atlas set (Gousias et al., 2012 and 2013). The cortical surface atlas was constructed by applying a kernel regression algorithm (Nadaraya et al., 1964) in the spectral domain. The surface atlas allowed the use of a joint spectral analysis to identify subject-to-subject cortical correspondences more accurately than the common spherical demon methods applied to voxel-based images. However, the developed atlas is not publicly available.

Xia et al. (2019) constructed an age-specific fetal cortical surface atlas from 25 reconstructed fetuses in healthy pregnancies. The atlas is defined for each week in the range of 26–29 GAs and consists of age-specific T2w template images equipped with biologically meaningful segmentation maps based on cortical growth patterns across vertices. Specifically, to comprehensively capture similarities of growth patterns among vertices, they first constructed two complementary similarity matrices (one based on growth trajectories of vertices, and the other based on the correlation profiles growth trajectories of vertices in relation to a set of reference points) and fused (Wang et al., 2014a) them to capture their common and complementary information. Finally, a spectral clustering (Ng et al., 2002) on the fused similarity matrix was performed to divide the spatiotemporal fetal cortical surface atlases into distinct regions grouped into 10 clusters: sensorimotor, posterior parietal, dorsolateral prefrontal, cingulate, and media frontal, ventrolateral prefrontal and anterior insula, orbitofrontal and anterior cingulate, medial temporal, posterior temporal, anterior temporal and posterior insula, occipital and precuneus. The final atlas was exploited to propose a new parcellation approach based on the dynamic changes on the cortical surface. It is not publicly available.

Karolis et al. (2023) introduced a spatio-temporal surface atlas from 242 normal fetal brains recruited in the dHCP project. The atlas is defined for each week in the range of 21–36 GAs and provides the pial, mid-thickness, white matter, and very inflated surfaces. Moreover, it provides some derived maps of changes in cortical morphology (thickness, curvature, and sulcal depth) and the result of the spherical registration to a template. The atlas was generated by adapting the procedure introduced for the neonatal surface atlas by Bozek et al. (2018), based on a spherical registration approach (Multimodal Surface Matching). Its generation involved three primary steps: first, a common reference space was initialized via affine sulcal-depth-based registration to the dHCP neonatal template at week 36; second, the template was coarsely refined using sulcal-depth-based nonlinear alignment; third, the template was refined using curvature-based alignment. This novel spatio-temporal atlas is the first publicly available surface atlas, opening new opportunities to explore fetal brain gyrification processes.

### 3.2. Fetal brain MRI datasets

Fetal brain MRI datasets, or multi-subject atlases, include as template images individual 3D reconstructions of a set of subjects (often derived from the T2w sequences) and their individual segmentation as label images. Datasets can be used as multi-subject atlases, enabling propagation of labels from the atlas to a new subject through a series of subject-to-subject registrations followed by a label fusion procedure. Furthermore, datasets are valuable resources that can be exploited to develop and benchmark new methods able to automatize labeling processes of the fetal brain by learning the complex relationship between distinct brain structures and inter-subject variability. Table 2 provides an overview of publicly available fetal brain MRI datasets.

**Fig. 9.**
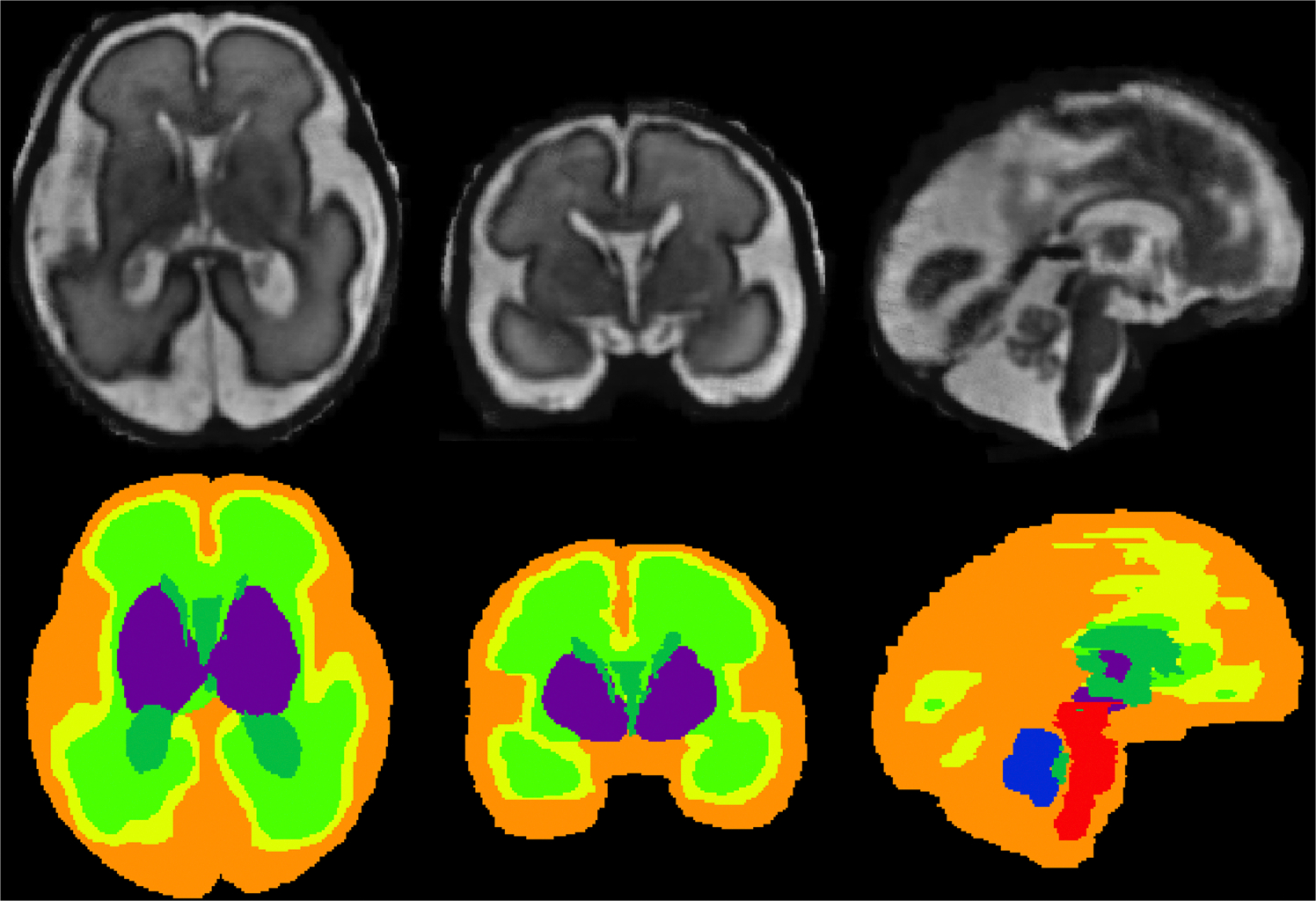
Example of manual segmentation (Orange: external cerebrospinal fluid; yellow: GM; bright green: WM; dark green: ventricles; blue: cerebellum; purple: deep GM: red: brainstem/spinal cord) on a 28.7-week fetus. Image adapted from Payette et al. (2023) publicly available dataset.

Payette et al. (2021) provided the first publicly available dataset (FeTA - Fetal Tissue Annotation and Segmentation Dataset) of 50 pathological (*N* = 32) and neurotypical (*N* = 18) MRI fetal brains with an age range from 20 to 33 GAs. Pathological subjects include fetuses with spina bifida who underwent an MRI either before or after surgery to repair the spinal lesion. The dataset includes 50 T2w fetal brain reconstructions (voxel size of 0.5 mm isotropic), each with a label image reporting manual segmentation of 7 different tissues/labels: external cerebrospinal fluid (eCSF), GM, WM, LV, CB, dGM and BS (Fig. 9). This dataset was introduced for the first time in the FeTA Challenge 2020, a multi-class and multi-centric image segmentation challenge, that aimed to develop generalizable automatic multi-class segmentation methods for developing human brain tissues.

New versions of this dataset were released at FeTA Challenge 2021 and 2022, respectively. The updated freely available dataset consists of 80 subjects (49 pathological and 31 neurotypical) in the range of 20–35 GAs (Payette et al., 2023). Similarly, each subject was released with a T2w fetal brain reconstruction (reconstructed with either the NiftyMIC (Ebner et al., 2020), MIALSRTK (Tourbier et al., 2015), SVRTK (Kuklisova et al., 2012)) and the corresponding seven label images (Fig. 9).

Urru et al. (2023) introduced a publicly available fetal dataset and a probabilistic atlas (see Section 3.1.1). The dataset ranges from 33 to 38 GAs and is specifically designed to aid in the segmentation process, exploiting the retro projection and label fusion approach (Avants et al., 2008; Wang et al., 2011). The dataset includes 20 T2w fetal brain reconstructions (voxel size of 0.86 mm isotropic), each with a label image reporting manual segmentation of 9 different tissues/labels obtained from an enhanced version of Gholipour et al. (2017) atlas: CSF, cGM, WM, LV, CB and BS, dGM, high-intensity WM, low-intensity WM.

## 4. Discussion

Fetal MRI is a third-level diagnostic tool for the characterization of the fetal central nervous system and is usually prescribed when routine ultrasound investigations are inconclusive (Pollatou et al., 2022). Since its application in clinical practice, fetal MRI has led to the development of new research topics on brain development. It provides a valuable characterization of the brain anatomy and functions (Oishi et al., 2019), leading to an increasing demand for normative atlases to be applied as references. Nowadays, technological progress, novel imaging techniques, and AI-based algorithms significantly boosted the generation of refined fetal brain atlases. 3T scanners spurred significant advancement in fetal MRI studies, notably supporting the acquisition of higher-quality images. Several concerns in the transition from 1.5T to 3T were related to the potential increase of the specific absorption rate (SAR), potential effects on the fetus growth or impact on the fetus auditory system development owing to the high noise level of the 3T magnets (Colleran et al., 2022). In their recent work, Manganaro et al. (2023) claim that there is no experimental evidence indicating negative effects on the fetus during 3T recording. For obvious reasons, both fetus and mother’s safety should be considered a priority. Thus, further studies should continue to investigate and definitely clarify this. Novel imaging techniques, with particular attention to DWI models such as the intravoxel incoherent motion (IVIM) model (Yuan et al., 2019; Jakab et al., 2018; Ercolani et al., 2021) and resting state fMRI (van den Heuvel et al., 2016; Vasung et al., 2019), have become crucial to better characterize -at a finer scale-the fetal brain anatomy and connectivity. AI-based algorithms have established valuable solutions for time-saving and precise acquisition (e.g., Singh et al., 2020), and reliable processing tools (e.g., Sobotka et al., 2022; Karimi et al., 2023). Furthermore, AI-based algorithms have already shown their potential in combining different imaging modalities (e.g., diffusion and structural MRI) (Ball et al., 2017). Therefore, generating multimodal spatiotemporal fetal brain atlases that combine multiple MRI-derived maps in the same anatomical space across the wider possible gestational age range is a future challenging trend for fetal brain research. Additional consideration is needed for multimodal atlas.

Similarly to the literature on adult population, the combination of functional and anatomical imaging approaches appears to be a future crucial direction in the fetal field to better characterize the close relationship between brain structures and functions (De Vareilles et al., 2023). However, several factors hinder the generation of multimodal atlases. One major factor is the technical challenge involved in acquiring and processing these types of data. For example, DTI approaches require longer acquisition time, making them very susceptible to the subject’s motion (Khan et al., 2019); fMRI approaches produce poor image resolution in both in and through-plane direction, which makes replicability of connectivity analysis difficult (Sobotka et al., 2022). Furthermore, the number of processing methods concerning more recent techniques (e.g., diffusion and functional MRI) is limited compared to the number of structural imaging methods (e.g., Uus et al., 2023c). Taken together, these considerations help in understanding why the use of multimodal atlases is challenging. A crucial additional point deserves to be considered: multimodal atlases should not be considered exclusively in terms of synergy between structural and functional approaches. For example, by combining distinct anatomical acquisition sequences, clinicians may significantly benefit from the use of different contrasts. Thus, multimodality should be considered both in terms of synergy between structural and functional data, and in terms of distinct contrasts of structural images.

In this study, we reviewed 10 structural anatomical atlases, 4 structural diffusion atlases, 4 surface atlases, and 3 datasets (or multi-subject atlases). Together with the atlas reference image, each of the publicly available anatomical atlases includes an atlas label image useful to identify and study specific brain structures (Table 1). From this point of view, we can identify two different approaches to the segmentation task. In the first approach, only the major brain structures are described (<10), such as the cortical GM or the ventricles. Other atlases provide a fine segmentation of each brain structure, identifying tens of regions for each major brain structure (>30) (Gholipour et al., 2017; Wu et al., 2021; Xu and Sun et al., 2022). This second approach may raise some concerns related to the fetal brain size and the acquired image resolution. The common acquisition protocol includes fast 2D T2w sequences with a voxel size of a high in-plane resolution of 0.4–1 mm, with a large slice thickness of 2–4 mm (Ciceri et al., 2023b), while the mean fetal brain volume ranges from 103 cm^3^ at 22–24 weeks (roughly a sphere with a diameter of 6.4 cm) to 319 cm^3^ at 32–34 weeks (Tran et al., 2023) (roughly a sphere with a radius of 9.2 cm). It is therefore crucial to maintain a good trade-off between resolution, reliability, and reproducibility when defining a segmentation scheme.

A crucial aspect to consider when dealing with fetal brain atlases is that the brain undergoes rapid and extensive changes during pregnancy. Thus, some structures may have different shapes in different GA periods, and some may be only temporary (i.e., detectable only in a specific GA range). Therefore, images from different GAs must be carefully combined when building a probabilistic template image. One common approach is to divide the original population into subsets on the basis of their GA and build a set of atlas reference images, each describing a short GA period (Habas et al., 2010; Clouchoux et al., 2012; Xia et al., 2019; Urru et al., 2023). Even if it is conceptually simple and effective, this method has the limit that each atlas reference image may be based only on a few subjects, and images referring to consecutive GA periods may be quite different. More sophisticated approaches include terms to account for the temporal evolution of the atlas reference images (e.g., kernel regression algorithms) (Serag et al., 2012; Dittrich et al., 2014; Wright et al., 2015; Khan et al., 2019; Gholipour et al., 2017; Xu and Sun et al., 2022; Chen et al., 2022), thus including all subjects in the atlas building procedure and ensuring temporal coherence among the resulting images. A further consequence is that region definition and segmentation protocols should be optimized for the GA range and reliable physiological segmentations may be largely different as the distance between their GA domain increases. For instance, in Gholipour et al. (2017), a different WM segmentation is provided for the ranges 21–30 GAs and 31–38 GAs.

Generally speaking, fetal atlas-based image analysis is characterized by a certain number of limitations, which risk generating inaccuracies in subsequent steps of analysis. Notably, subject-atlas registration errors may result in altered reconstruction of anatomical structures (mainly in regions of high anatomical complexity). Single-subject atlas-based analysis may not be able to accurately represent anatomy across individual development, inter-subject variability, or population cohorts. In addition, the atlas may not accurately represent the altered anatomy of atypical brain development. Analysis of low-resolution images may lead to loss of fine anatomical details or ambiguous tissue boundaries. Therefore, to mitigate these pitfalls, researchers should consider proper pre-processing pipelines and atlases for specific cohorts. The combined use of distinct probabilistic atlases may be a valuable solution to assess accuracy and reliability of results. Alternative approaches, such as data-driven methods, have also been explored (Payette et al., 2023; Fidon et al., 2024). In this scenario, Deep Learning (DL) methods are rapidly gaining ground. DL methods try to avoid these pitfalls by learning the complex relationship between distinct brain structures directly from the subject training data, rather than inferring it from an atlas. However, DL methods require large datasets to be trained with, thus atlas-based approaches continue to be considered important in some scenarios, for example when explicit anatomical priors or specialized knowledge are available, or with small datasets. Thus, in the light of the current complex scenario, a combination of DL and atlas-based methods can be a pragmatic way to exploit the strengths of both approaches (Litjens et al., 2017; Fidon et al., 2024).

The 71.4% of reviewed atlases in the fetal context are publicly available. This is a great achievement, considering data privacy requirements, and also general concerns related to the peculiarity of this population. Public atlases and datasets play a pivotal role in advancing replicable research, improving clinical applications, and more generally fostering the medical imaging domain. Initiatives such as international projects (e.g., dHCP), and challenges (e.g., FeTA Challenge) aim to facilitate sharing of medical imaging data for research purposes and are valuable resources for developing and benchmarking new analysis methods and algorithms. Despite this is being a remarkable achievement in terms of open science, the scenario is more complex for clinical cases. Only one clinical atlas (Fidon et al., 2022) and one clinical dataset (Payette et al., 2023) are publicly available (both of them related to the SBA condition). One of the main concerns for fetal brain disease-specific atlases and datasets is related to the generally low incidence of disease compared to the general population. Heterogeneity in clinical presentation and complexity of disease-related feature annotation are further concerns. Last but not least, data privacy regulation in clinical populations is - for obvious reasons - generally more restrictive.

### 4.1. Clinical, research, and ethical implications

Prenatal brain imaging in humans is a fascinating, yet extremely complex, challenge. Fetal brain atlases and datasets support monitoring of brain development milestones, identification of abnormalities and in turn they offer new critical insights for prodromic signs of potential (future) clinical conditions. First of all, the human brain is characterized by an intricate pattern of gyri and sulci. Despite considerable efforts in the field, neurobiological mechanisms resulting in gyrification remain far from being fully understood. This is not to be overlooked, considering that atypical gyrification trajectories in prenatal development have been associated with a number of neurological, neurocognitive, and behavioral disorders. Advanced multimodal MRI techniques in fetal imaging are providing previously unexplored information about prenatal brain morphological architecture, and functional connectivity (De Asis-Cruz and Limperopoulos, 2023). Prenatal imaging is a new powerful tool contributing to the definition of normative patterns of fetal brain development that may support identification of high-risk fetuses (Xu et al. 2020; Machado-Rivas et al. 2022; Kim et al. 2023). In turn, potential associations between advanced prenatal MRI brain findings and long-term neurodevelopmental outcomes may play an important role in promoting (very) early postnatal habilitative/rehabilitative interventions that could limit potential negative sequela in adulthood.

In the current work, the cerebellum, among other structures, deserves a brief mention. Nowadays, a compelling body of studies agrees on the critical role of the cerebellum for both motor and non-motor functions (Buckner et al., 2013; Marek et al., 2018; Diedrichsen et al., 2019). In addition, it has been hypothesized that a primary injury to the cerebellum may result in a secondary dysfunction in functionally connected cerebral regions (Wang et al., 2014; Casartelli et al. 2018). Interestingly for our aims, the human cerebellum has a protracted developmental trajectory compared to neocortical structures (Wang et al. 2001; Sathyanesan et al. 2019). Thus, it also has a large critical time window of vulnerability (De Asis-Cruz and Limperopoulos, 2023), and prenatal imaging advancements may provide a promising perspective to clinically monitor these aspects. Notably, the cerebellum was considered in all anatomical atlases and datasets that have been published in the past 5 years; this indirectly supports the growing interest for this specific anatomical structure.

Undoubtedly current progress in prenatal neuroimaging techniques will provide important insights into fetal brain research and may also provide significant support in certain clinical practices such as prenatal development monitoring (see for example, Zoetmulder et al. 2023; Stuempflen et al. 2023). This point can be shared despite clear difficulties in applying prenatal neuroimaging in routine clinical practice. As many other cutting-edge and pioneering techniques in biomedical domains (e.g., brain-computer interface; neuroprosthetics; etc.), prenatal neuroimaging is confined to clinical centers implement appropriate safety procedures and data collection, processing, and interpretation. In contrast, ethical implications of prenatal imaging advancement are seemingly often underestimated. We are aware of the significant and urgent nature of these ethical issues, but their evaluation probably goes beyond our specific aims in the present work. We urge basic researchers, clinicians, caregivers, public health decision-makers, and notably the general population to consider the ethical implications of scientific advancement in this field. The risk of neglecting “new” and substantially unexplored ethical issues is not limited to the advancement in prenatal neuroimaging. It is becoming a fundamental concern for everyone dealing with AI, regardless of the specific (sub-)field. For example, an AI-based approach in prenatal brain imaging becomes extremely (and potentially dramatically) important when dealing with the possibility that clinicians can request other more invasive in-utero investigations.

We should go back to considering technical advancement a tool for human progress, and not misleadingly the goal of human progress. Ethical concerns would thus regain their central place in the scientific endeavor. This will not automatically address concerns but it will at least promote a more critically oriented approach.

## 5. Conclusion

Prenatal brain is a critical window for individual development. The investigation of early stages of development should be considered a priority by the scientific community to maximize our understanding of the developing brain.

In this work, we provided a systematic review of fetal brain atlases and datasets. We proposed a terminological clarification of specific terms often inconsistently employed in the literature and then we specifically focused on the prenatal period, analyzing 18 fetal brain atlases and 3 datasets. Atlas-based approaches still play a crucial role as repository of knowledge about the fetal brain architecture across GAs. However, datasets surely are a promising domain in this field, supporting cutting-edge AI-based approaches.

The growing body of studies focusing on the prenatal brain development certifies the critical role that atlases and datasets reviewed in this work can assume for clinical and research aims. Beyond it, we stress the need of pursuing in monitoring issues concerning safety, feasibility, reliability, and ethical concerns related to fetal brain research.

## Supplementary Material

SM

## Figures and Tables

**Table 1:** Publicly available atlases of fetal brain from MR images.

Atlases	Template	Isotropic resolution	Regions of interest	Time-points	Original cohort	Public link

**Anatomical**						
Habas et al. (2010)	T2w	0.5 mm	4	21–24 weeks	20	http://depts.washington.edu/bicg/research/fba.php
Serag et al. (2012)	T2w	0.86 mm	4	23–37 weeks	80	https://brain-development.org/brain-atlases/fetal-brain-atlases/fetal-brain-atlas-serag
Gholipour et al. (2017)	T2w	0.8 mm	124	21–38 weeks	81	http://crl.med.harvard.edu/research/fetal_brain_atlas
Wu et al. (2021)	T2w	0.8 mm	124	22–35 weeks	89	https://github.com/DeepBMI/FBA-Chinese
Fidon et al. (2022)	T2w	0.8 mm	8	21–34 weeks	37	https://www.synapse.org/#!Synapse:syn25887675/wiki/611424
Xu et al. (2022)	T2w	0.8 mm	85	23–38 weeks	90	https://github.com/Thea-Eddie-Amy/CHN-fetal-brain-atlas
Urru et al. (2023) ^	T2w	0.86 mm	9	21–38 weeks	81	https://github.com/urrand/perinatal-pipeline
Uus et al. (2023)	T2w, T1w	0.5 mm	19	21–36 weeks	187	https://gin.g-node.org/kcl_cdb/fetal_brain_mri_atlas/src/master
**DWI**						
Khan et al. (2019)	FA, cFA, MD	0.75 mm	-	22–38 weeks	67	http://crl.med.harvard.edu/research/fetal_brain_atlas
Chen et al. (2022)	tensor, FA, cFA, MD, FOD*, RD, AD	1.2 mm	-	24–38 weeks	89	https://github.com/RuikeChen/Fetal-Brain-dMRI-Atlas
Uus et al. (2023)	DWI, FA, MD, RD, FOD	0.5 mm	19	21–36 weeks	187	https://gin.g-node.org/kcl_cdb/fetal_brain_mri_atlas/src/master
**Surface**						
Karolis et al. (2023)	pial, mid-thickness, WM and vinflated surfaces	-	thickness, curvature, sulcal depth	21–36 weeks	242	https://doi.org/10.12751/g-node.qj5hs7

^ Gholipour et al. (2017) new version;

* present only at week 24,25 and 26.

**Table 2:** Publicly available datasets of fetal brain from MR images.

Dataset	Individual template	Isotropic resolution	Regions of interest	GAs range	Original cohort	Public link

Payette et al. (2021)	T2w	0.5 mm	7	20–34 weeks	50	https://www.synapse.org/#!Synapse:syn23747212/wiki/608434
Payette et al. (2023)	T2w	0.5 mm	7	20–35 weeks	80	https://www.synapse.org/#!Synapse:syn25649159/wiki/610007
Urru et al. (2023)	T2w	0.86 mm	9	33–38 weeks	20	https://github.com/urrand/perinatal-pipeline

## Data Availability

No data was used for the research described in the article.

## Footnotes

1
https://github.com/gift-surg/NiftyMIC

2
https://github.com/Medical-Image-Analysis-Laboratory/mialsuperresolutiontoolkit

3
https://github.com/SVRTK/svrtk-docker-gpu

4
https://github.com/daviddmc/NeSVoR

## Abbreviations:

T2W: T2-weighted
GA: gestational age
MRI: magnetic resonance imaging
DL: deep learning
ROI: region of interest
CB: cerebellum
WM: white matter
GM: gray matter
CGM: cortical gray matter
DGM: deep gray matter
LV: lateral ventricles
BS: brain stem
CSF: cerebrospinal fluid
ECSF: external cerebrospinal fluid
VCSF: ventricular cerebrospinal fluid
CP: cortical plate
GE: ganglionic eminence
CC: corpus callosum
HP: hippocampus
LN: lentiform nucleus
TH: thalamus
DWI: diffusion weighted imaging
DTI: diffusion tensor imaging
FA: fractional anisotropy
MD: mean diffusivity
CFA: color-coded fractional anisotropy
RD: radial diffusivity
AD: axial diffusivity
FOD: fiber orientation distribution
