# Supplementary material for: Fetal brain MRI atlases and datasets: A review
Source: Neuroimage. Author manuscript; Available in PMC 2025 May 9. (PMC12064217; doi:10.1016/j.neuroimage.2024.120603)
Supplement: SM [file NIHMS2074594-supplement-SM.docx]

**Supplementary File**

Search in PubMed, performed on January 2024:

| # | Search Terms | Results |
| --- | --- | --- |
| 1 | "fetal"[Title/Abstract] OR "fetus"[Title/Abstract] OR "embryo"[Title/Abstract] OR "embryonal"[Title/Abstract] OR "intrauterine"[Title/Abstract] OR "antepartum"[Title/Abstract] OR “prenatal”[Title/Abstract] | 577,806 |
| 2 | “atlas"[Title/Abstract] OR "template"[Title/Abstract] OR "dataset"[Title/Abstract] | 260,046 |
| 3 | "MRI"[Title/Abstract] OR "imaging"[Title/Abstract] OR "radiology"[Title/Abstract] | 1,269,059 |
| 4 | "brain"[Title/Abstract] OR "cerebrum"[Title/Abstract] | 1,231,024 |
| 5 | #1 AND #2 AND #3 AND #4 AND (English[Filter])) | 144 |
